# Neurobiological mechanisms associated with antipsychotic drug-induced dystonia

**DOI:** 10.1177/0269881120944156

**Published:** 2020-09-09

**Authors:** Anton JM Loonen, Svetlana A Ivanova

**Affiliations:** 1 Groningen Research Institute of Pharmacy, Pharmacotherapy, -Epidemiology and -Economics, University of Groningen, Groningen, The Netherlands; 2 Geestelijke GezondheidsZorg Westelijk Noord-Brabant (GGZ WNB), Mental Health Hospital, Halsteren, The Netherlands; 3 Mental Health Research Institute, Tomsk National Research Medical Center of the Russian Academy of Sciences, Tomsk, Russian Federation; 4 National Research Tomsk Polytechnic University, Tomsk, Russian Federation; 5 Siberian State Medical University, Tomsk, Russian Federation

**Keywords:** Dystonia, antipsychotics, extrapyramidal side effects, cholinergic interneurons, neuroplasticity

## Abstract

Dystonia is by far the most intrusive and invalidating extrapyramidal side effect of potent classical antipsychotic drugs. Antipsychotic drug-induced dystonia is classified in both acute and tardive forms. The incidence of drug-induced dystonia is associated with the affinity to inhibitory dopamine D2 receptors. Particularly acute dystonia can be treated with anticholinergic drugs, but the tardive form may also respond to such antimuscarinic treatment, which contrasts their effects in tardive dyskinesia. Combining knowledge of the pathophysiology of primary focal dystonia with the anatomical and pharmacological organization of the extrapyramidal system may shed some light on the mechanism of antipsychotic drug-induced dystonia. A suitable hypothesis is derived from the understanding that focal dystonia may be due to a faulty processing of somatosensory input, so leading to inappropriate execution of well-trained motor programmes. Neuroplastic alterations of the sensitivity of extrapyramidal medium-sized spiny projection neurons to stimulation, which are induced by the training of specific complex movements, lead to the sophisticated execution of these motor plans. The sudden and non-selective disinhibition of indirect pathway medium-sized spiny projection neurons by blocking dopamine D2 receptors may distort this process. Shutting down the widespread influence of tonically active giant cholinergic interneurons on all medium-sized spiny projection neurons by blocking muscarinic receptors may result in a reduction of the influence of extrapyramidal cortical-striatal-thalamic-cortical regulation. Furthermore, striatal cholinergic interneurons have an important role to play in integrating cerebellar input with the output of cerebral cortex, and are also targeted by dopaminergic nigrostriatal fibres affecting dopamine D2 receptors.

## Introduction

Dystonia is a movement disorder characterised by sustained or intermittent muscle contractions of muscle antagonists causing relatively slow, twisting movements and often leading to abnormal postures (Albanese et al., 2019; Jameson et al., 2018; Loonen and van Praag, 2007). The term ‘athetosis’ generally denotes abnormal movements that are slow, sinuous, and writhing in character. When the movements are so sustained that they are better regarded as abnormal postures, the term ‘dystonia’ is used, but many now prefer the use of dystonic postures as well as movements to describe the spectrum of clinical features (Simon et al., 2017). The abnormal movements and postures may be generalised (involving the trunk and at least two other sites) or restricted in distribution, such as to the neck (torticollis), hand and forearm (writer’s cramp), or mouth (oromandibular dystonia) (Albanese et al., 2019; Simon et al., 2017). Dystonia is often initiated or worsened by voluntary action and associated with overflow muscle activation (Jameson et al., 2018). Sometimes, focal dystonia is task-specific and can manifest itself as a loss of voluntary motor control in extensively trained movements such as writing or playing a musical instrument (Altenmüller and Müller, 2013). Some of the most interesting phenomena accompanying some focal dystonic disorders are the different types of alleviating manoeuvres (‘gestes antagonistes’ or ‘sensory tricks’) (Mailankody and Pal, 2017; Ochudlo et al., 2007; Patel et al., 2014; Ramos et al., 2014). Their presence has been confirmed in many forms of focal dystonias, for example in cervical dystonia, where a geste antagoniste is an extraordinary clinical feature that attenuates disease symptoms by slight touch to a specific area of the face or head (Filip et al., 2016; Ramos et al., 2014).

Dystonia can be drug-induced; it has resulted from administration of dopamine agonists, lithium, serotonin reuptake inhibitors, carbamazepine and metoclopramide, but is commonly known as a complication of treatment by potent dopamine receptor antagonists when used as an antipsychotic agent (Jain, 2012; Simon et al., 2017). Together with akathisia (Loonen and Stahl, 2011), Parkinsonism and dyskinesia (Loonen and Ivanova, 2013), drug-induced dystonia is considered to be one of the primary extrapyramidal side effects of first and second generation antipsychotic drugs (Caroff and Campbell 2016; Pierre, 2005) (see Tables 1 and 2). Specific dystonic phenotypes which are more often drug-induced are the so-called ‘rabbit’ (Catena et al., 2007; Schwartz and Hocherman, 2004), ‘tongue protrusion/retraction’ (Porsolt et al., 2013) and ‘Pisa’ syndromes (Lee, 2018; Suzuki and Matsuzaka, 2002).

**Table 1.** Pharmacological characteristics of ‘drugs for psychosis’ (antipsychotic drugs) applying NbN systematics.

Indication-based terminology	Pharmacology	Mode of action	Drugs
Typical (1^st^ generation) antipsychotics	Dopamine	Receptor antagonist (D2-type) Receptor antagonist (D1-type)	Particularly: high-potency drugs Particularly: flupenthixol, zuclopenthixol, fluphenazine
	Dopamine, serotonin	Receptor antagonist (HTR2A) Receptor antagonist (HTR2C)	Pipamperone Pipamperone
	Dopamine, acetylcholine	Receptor antagonist (CHRM) Receptor antagonist (CHRN)	Particularly: thioridazine, chlorprothixene None
Atypical (2^nd^ generation) antipsychotics	Dopamine	Receptor antagonist (D2) Receptor antagonist (D1)	Low affinity: quetiapine, sulpiride Particularly: asenapine, sertindole, olanzapine
	Dopamine, serotonin	Receptor antagonism (HTR2A) Receptor antagonism (HTR2C) Inverse receptor agonism (HTR2C)	Low: amisulpride, sulpiride Particularly: clozapine, olanzapine Particularly: clozapine
	Dopamine, acetylcholine	Receptor antagonist (CHRM) Inverse receptor agonism (CHRM4) Receptor antagonist (CHRN)	Particularly: clozapine, olanzapine Particularly: clozapine None

**Table 2.** Receptor interactions possibly contributing to the mechanism of dystonia.

Receptor antagonism	Site	Effect
Dopamine D2-type (DRD2, DRD3, DRD4)	Indirect pathway MSNs	Mismatch of the activity of direct and indirect pathways Mismatch of the activity of convergent extrapyramidal circuits
	Striatal cholinergic interneurons	Widespread inhibition of ACh release
	Dopaminergic terminals targeting FSIs	Less activation of inhibitory FSIs by DRD5
Muscarinic ACh M1 class (CHRM1, CHRM3, CHRM5)	Direct and indirect pathway	Equal activation of direct and indirect pathways
Muscarinic ACh M2 class (CHRM2, CHRM4)	Striatal cholinergic interneurons GABA-ergic interneurons Glutamatergic terminals Direct pathway MSNs	Widespread inhibition of ACh release Mixed effects Nonspecific inhibition of direct and indirect pathways Selective inhibition of direct pathway
Nicotinic ACh receptors	Ascending dopaminergic pathways Cholinergic interneurons GABA-ergic interneurons Glutamatergic terminals Dopaminergic terminals	Nonspecific striatal D1 and D2 activation Widespread increase of ACh release Mixed effects Nonspecific activation of direct and indirect pathways Nonspecific activation of direct and indirect pathways

ACh: acetylcholine; FSI: fast-spiking GABA-ergic interneuron; GABA: gamma-amino butyric acid; MSN: medium spiny neuron.

Acute dystonia or dyskinesia (such as blepharospasm, torticollis, or facial grimacing) is an occasional complication of dopamine receptor antagonist treatment, in 95% of all cases occurring within 96 h of starting treatment with an antipsychotic drug or after a substantial augmentation of its dose (van Harten et al., 1999). According to the first survey known to us, which is based on 3775 patients, they are thought to be related to a rapid and intense change of dopamine D2 receptor antagonism because they appear ‘earlier in time and with progressively smaller doses of the compound as one ascends the potency scale from chlorpromazine to fluphenazine’ (i.e. when dopamine D2 affinity is higher) (Ayd Jr, 1961). This is particularly true if the drug is administered intramuscularly or in suppository form (Ayd Jr., 1961). Men and younger patients show increased susceptibility to this complication (Ayd Jr, 1961). Recent cocaine abuse is a major risk factor (van Harten et al., 1998). The pathophysiologic basis of the disturbance is unclear, but parenteral treatment with an anticholinergic drug usually alleviates it (Simon et al., 2017; van Harten et al., 1999). Tardive dystonia is usually segmental in distribution, affecting two or more contiguous body parts, such as the face and neck or arm and trunk. It is less often focal: when this is the case, the head and neck in particular are most likely to be affected, producing blepharospasm, torticollis, or oromandibular dystonia. Generalised dystonia is least common and tends to occur in younger patients. Treatment is as for tardive dyskinesia, except that anticholinergic drugs may also be helpful; focal dystonias may also respond to local injection of botulinum A toxin (Adityanjee et al., 1999; Simon et al., 2017; van Harten and Kahn; 1999). Studies on the treatment effects of the Vesicular Monoamine Transporter 2 (VMAT-2) inhibitors, valbenazine and deutetrabenazine, have shown that they are effective in treating tardive dyskinesia (Bhidayasiri et al., 2018; Solmi et al., 2018), but studies specifically addressing tardive dystonia are still lacking. It is a pity that a specifically designed instrument to carefully measure several movement disorders simultaneously (Loonen et al., 2000; Loonen et al., 2001) was not used in these trials, because this could have indicated possible efficacy in tardive dystonia. Studies with these or similar suitable instruments (Loonen and van Praag, 2007) addressing the therapeutic effects of VMAT-2 inhibitors in patients with schizophrenia who have been exposed to antipsychotic drug treatment for at least three months (applying criteria of Schooler and Kane, 1982) and show symptoms of dyskinesia and/or dystonia are urgently needed. These controlled trials should distinguish between the effects on orofacial (classical) and limb-truncal (peripheral) dyskinesia as the last form of dyskinesia may be more close related to dystonia than the first one (Loonen et al., 2019).

Apart from aforementioned drug treatments, neurosurgical interventions, in particular deep brain stimulation (DBS), can be considered as treatment options for tardive movement disorders (Blomstedt et al., 2009; Marcerollo and Deuschl, 2018). Abnormal neural oscillations within the extrapyramidal circuit can be applied as biomarker within the context of adaptive DBS (Piña-Fuentes et al., 2018).

In the present article we will describe a model for the pathophysiology of acute and tardive drug-induced dystonia. We start with a description of the extrapyramidal system and the role of muscarinic receptors in modifying its functioning.

## The human extrapyramidal system

The neuronal circuit which is covered by the motoric extrapyramidal subsystem regulates the velocity and amplitude of voluntary movements (Loonen and Ivanova, 2013). This function is specifically addressed by a cortical-striatal-thalamic-cortical (CSTC) circuit containing the putamen as the entry station to the basal ganglia (Loonen and Ivanova, 2013). However, it should be realised that this motoric extrapyramidal system is part of a general subcortical regulatory system which has existed in almost unchanged form for more than half a billion years (Grillner et al., 2013; Grillner and Robertson, 2016; Robertson et al., 2014). We have recently described that the ‘extrapyramidal system’ actually consists of three parallel divisions: a ventral and a dorsal ‘extrapyramidal’ as well as an ‘amygdaloid’ section (Loonen and Ivanova, 2018a; 2018b). Distinguishing this last section results from the understanding that the most ancient vertebrate animals living about 560 million years ago (mya) already had a forebrain with a modern extrapyramidal system consisting of similar components as nowadays found in humans (Loonen and Ivanova, 2015; 2016). Similar to the extrapyramidal system of our earliest vertebrate ancestors, the human amygdaloid system initiates the fundamental, intuitive appetitive, reproductive and defensive behaviours which are necessary to maintain life and to have offspring. In more recent amphibian-like ancestors (370 mya), this primary striatopallidum is represented by the central and medial amygdala (striatal part) and bed nucleus of the stria terminalis (pallidal part), and a ventral striatopallidum had evolved as component of a future ventral extrapyramidal system (Loonen and Ivanova, 2016). This secondary extrapyramidal system, which includes the nucleus accumbens in humans, regulates the intensity of the essential behaviours initiated by the amygdaloid extrapyramidal system. The intensity corresponds to motivation to execute reward-bringing and distress-avoiding behaviours. In early mammals (145 mya), the cerebral neocortex with a corresponding dorsal extrapyramidal system started to evolve. This tertiary striatopallidum consists of caudate nucleus, putamen and globus pallidus and regulates rational, voluntary behaviour (Loonen and Ivanova, 2018a, 2018b, 2019). In the following, we will concentrate on the structure of this latter part (see Loonen and Ivanova, 2013), but it is important to realise that the dorsal extrapyramidal system can only function in mutual interaction with the ventral and amygdaloid parts of this system. This might, for example, explain the phenomenological similarities and the relationship between anxiety symptoms, akathisia, dyskinesia and dystonia.

**Figure 1.**
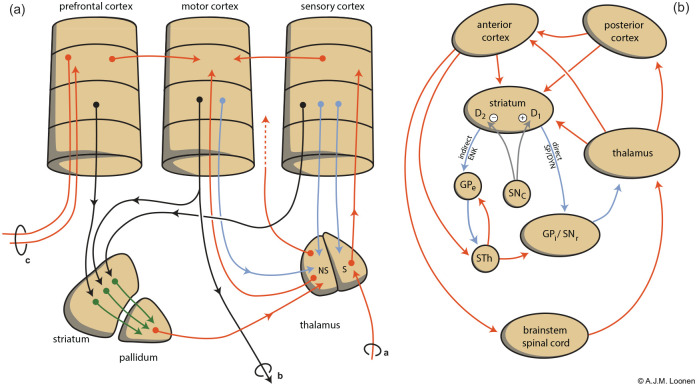
Schematic representation of the extrapyramidal cortico-striatal-[...]-thalamic-cortical (CSTC) system (Loonen and Ivanova, 2013). (a) Converging nature of parallel extrapyramidal circuits. Different parts of the cerebral cortex are linked to the motor cortex by intracortical connections, but also via a set of separate CSTC circuits which converge within the relevant part of the striatopallidal basal ganglia. Note the existence of a re-entry circuit starting and ending with the motor cortex. In (a) letter a indicates somatosensory input, b indicates motor output, c indicates output to the contralateral cerebral cortex. Colour of the arrows is insignificant. (b) Indirect (left) and direct (right) striatopallidal pathways representing [...] of the CSTC circuits. Corticostriatal neurons synapse with two types of inhibitory medium spiny neurons (MSNs): D2 receptor-carrying, enkephalin-containing, indirect pathway MSNs and D1 receptor-carrying, substance P and dynorphin-containing, direct pathway MSNs. Stimulation of both D1 and D2 receptors results in disinhibition of thalamocortical neurons and therefore increased cortical output. Red: stimulatory, blue: inhibitory. D1: dopamine D1 receptor-expressing medium spiny neurons D2: dopamine D2 receptor-expressing medium spiny neuron; GPe: globus pallidum externa; GPi: globus pallidus interna; NS: non-specific; S: specific; SNc: substantia nigra pars compacta; SNr: substantia nigra pars reticulata; STh: nucleus subthalamicus.

Every CSTC circuit consists of a direct and indirect pathway (Figure 1(b)) (Loonen and Ivanova 2013). The putamen and caudate nucleus receive input by excitatory glutamatergic corticostriatal fibres, which stimulate gamma-amino butyric acid (GABA)-ergic medium spiny neurons (MSNs). Two types of MSN exist, constituting the first fragment of the afore-mentioned direct and indirect pathway to the globus pallidus interna (GPi)/substantia nigra pars reticulata (SNr) (Figure 1(b)). In humans, the main output of the basal ganglia flows via the thalamus to the frontal cerebral cortex, but other output channels include upper brainstem nuclei (Nieuwenhuys et al., 2008). In Parkinsonian patients, an increase in neuronal activities in the GPi/SNr results in an inhibition of neurons of the thalamus and brainstem causing akinesia and rigidity, respectively (Kato, 1997). Theoretically this increase can produce dystonia with abnormal postures by maladjusted muscle contractions as well as inappropriate muscle tone. This augmented activity can be due to a reduced stimulation of excitatory dopamine D1 receptors or by decreased stimulation of inhibitory dopamine D2 receptors on MSNs of the direct and indirect extrapyramidal pathway, respectively. Antipsychotic drugs cause extrapyramidal symptoms mainly by antagonising dopamine D2 receptors.

The mammalian extrapyramidal system is also, largely, a converging CSTC circuit (Figure 1(a)) (Loonen and Ivanova, 2013, 2018b). The cerebral cortex is connected with the input ganglia of the extrapyramidal system in a (more or less) topographically arranged fashion (Alexander et al., 1986; Heimer, 2003; Voogd et al., 1998). This is important in order to have the capability of adapting the velocity and magnitude of the contractions of different involved muscles whilst executing a specific voluntary movement. By adjusting the sensitivity of direct and indirect pathway MSNs for stimulation by corticostriatal and thalamostriatal synapses, the putamen can learn how separate collaborating muscles should precisely contract in order to execute a complex movement of the individual. With training, the sensitivity of glutamatergic synapses can be changed by long-term potentiation (LTP) and long-term depression (LTD) (Calabresi et al. 2007; Loonen and Ivanova, 2013; Lovinger, 2010; Perrin and Venance, 2019). This could explain procedural memory formation within the basal ganglia. Inappropriate adjustment of the magnitude and/or velocity of contractions of collaborating muscle group would induce dystonic movements.

The (dorsal) striatum is inhomogeneous (Loonen et al., 2019). It consists of a continuous matrix with unevenly distributed striosomes or patches; these embedded striosomes decrease along the anterior-posterior and medial-lateral axis (Brimblecombe and Cragg, 2017; Kreutzer, 2009). The corticostriatal fibres of the aforementioned CSTC circuits target the matrix component, but the striosomes receive input from (limbic) medial prefrontal cortical areas and corticoid parts of the amygdaloid complex (Eblen and Graybiel, 1995; Ragsdale and Graybiel, 1988). This striosomal compartment regulates the activity of ascending dopaminergic neurons of midbrain centres in response to relevant stimuli (Friedman et al., 2015; Loonen et al., 2019). We have suggested that genetic differences between classical (orofacial) and peripheral (limb-truncal) dyskinesia are related to this striosome-matrix diversity (Loonen et al., 2019). The same could, *mutatis mutandis*, be true for dystonia as limb-truncal manifestations are dominant here; classical dyskinesia could primarily be related to the dysfunction of the striosomal compartment and dystonia to the matrix.

## Cholinergic receptors

The vast majority of the neurons of the corpus striatum (caudate, putamen, accumbens) belong to GABA-ergic MSNs (∼95%) or to three types of medium-sized aspiny interneurons (∼4%). The latter interneurons are also often said to be GABA-ergic, but one of these types – interneurons, which produce nitric oxide (NO) – do actually not express the GABA synthetising enzyme glutamic acid decarboxylase (Centonze et al., 2003a; Cicchetti et al., 2000). Only about 1–2% of the striatal nerve cells are cholinergic interneurons, but still, these giant, aspiny neurons make the striatum the largest cholinergic nucleus of the central nervous system as they ramify extensively and send projections widely throughout this ganglion (Gonzales and Smith, 2015; Lim et al., 2014). Almost all striatal cholinergic fibres belong to one of these cholinergic interneurons, which are believed to be the analogues of tonically active neurons (TANs) identified by in vivo recordings in the putamen of primates (Apicella, 2017; Deffains and Bergman, 2015). This means that these neurons show spontaneous activity, in which this basal activity can be modulated upwards and downwards by synaptic input. However, part of the cholinergic input to the striatum originates within brainstem cholinergic nuclei – i.e. the pedunculopontine (PPN) and laterodorsal tegmental (LDT) nuclei – which also modulate striatal activity by affecting ascending dopaminergic and thalamostriatal neurons (Dautan et al., 2014). Acetylcholine stimulates both striatal nicotinic and muscarinic receptors. Muscarinic receptors are more widely spread and can be divided in excitatory M1-class (M1, M3, M5) and inhibitory M2-class (M2, M4) receptors (Goldberg et al., 2012; Lim et al., 2014).

Nicotinic acetylcholine receptors (nAChRs) in the brain are widely expressed pentameric ion channels which have an excitatory effect upon activation (Loonen, 2013; Zoli et al., 2015). nAChRs are particularly abundant within the medial division of the habenuloid complex (Batalla et al., 2017; Loonen et al., 2017) which controls distress-avoiding behaviour by affecting midbrain nuclei, and also directly increase excitatory input to ascending dopaminergic midbrain neurons (de Kloet et al., 2015) which modulate the intensity of reward-seeking behaviour. Both mechanisms regulate ascending monoaminergic input to the striatum by upper brainstem neurons. Within the striatum nAChRs are expressed by cholinergic and GABA-ergic interneurons (English et al., 2011; Lim et al., 2014) as well as by dopaminergic and glutamatergic terminals (Kreutzer, 2009; Lim et al., 2014; Loonen et al., 2019). GABA-ergic projection neurons, MSNs, express nicotinic receptors only to a very limited extent (Bordia and Perez, 2019; Loonen et al., 2019). The influence of nAChRs on neurotransmitter release from glutamatergic and dopaminergic terminals suggests a possible role in the pathogenesis and treatment of certain movement disorders (particularly levodopa-induced dyskinesia); this is supported by available evidence (Quik et al. 2019). The effects of drugs antagonising muscarinic receptors, however, largely surpass those of substances which affect nAChRs and are therefore considered in this article in more detail.

Muscarinic receptors belong to a family of about 950 G protein-coupled receptors (GPCRs). Four types of G proteins exist, i.e. Gs, Gi, Go and Gt (apart from Gq, which is discussed later). All these G proteins are composed of three sub-units as an αGDPβγ trimer in inactive form. The activity is determined by the dissociated αGTP and βγ subunits. The G proteins are generally classified according to their α subunits into Gs (activates adenylate cyclase), Gi and Go (inhibits adenylate cyclase, and Gt (activates cGMP-phosphodiesterase). The βγ subunits are also involved in activation or inhibition of adenylate cyclase, and also in other functions. Based on present knowledge about the brain, it is thought that M2 and M4 receptors interact with Gi-, Go- and Gn-type G protein (all inhibitory vs adenylate cyclase). Another type of G protein links muscarinic receptors to the production of the second messengers diacylglycerol and inositol triphosphate (IP3), i.e. Gq-type G proteins. Muscarinic acetylcholine receptors M1, M3 and M5 are coupled to Gq-type G proteins.

**Figure 2.**
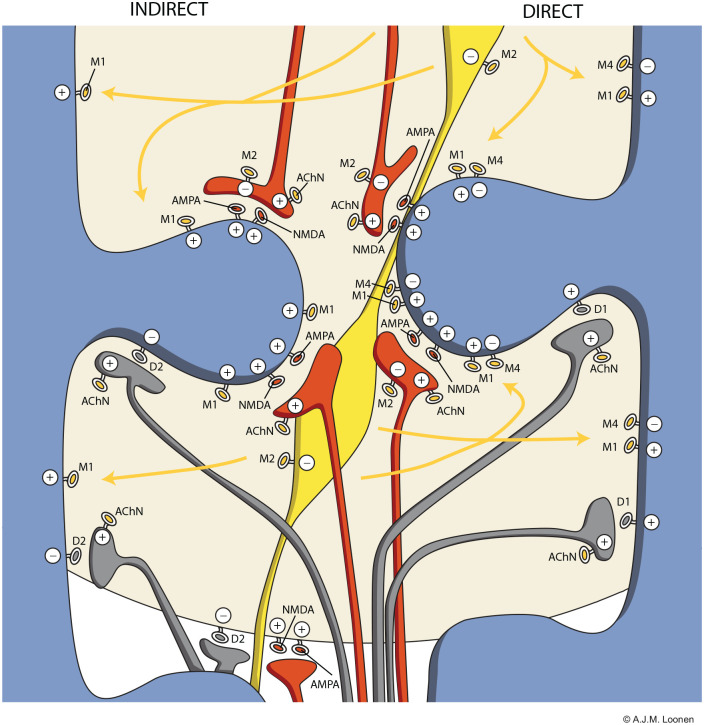
Distribution of cholinergic neurons within the striatum. Indirect pathway medium spiny neurons (left) carry few M4-type muscarinic acetylcholine receptors in comparison to direct pathway medium spiny neurons (right). Large and extensively ramified cholinergic interneurons release acetylcholine freely from varicosities and consequently affect a substantial striatal area. Red: glutamatergic terminals (upper = corticostriatal and lower = thalamostriatal); blue: dendrites of gamma-amino butyric acid (GABA)-ergic medium spiny neurons; yellow: cell body and varicosities of cholinergic interneurons; grey: dopaminergic synapses. AChN: nicotinic acetylcholine receptor; AMPA: α-amino-3-hydroxy-5-methyl-4-isoxazolepropionate glutamatergic receptor; D2: D2-type dopaminergic receptor; M1–M4: M1–M4 type muscarinic acetylcholine receptors; NMDA: N-methyl-D-aspartate glutamatergic receptor.

A simplified representation of the connectivity of cholinergic interneurons is shown in Figure 2 (adapted from Loonen et al., 2017). Cholinergic interneurons receive neuronal input from a variety of intrastriatal and extrastriatal nerve cells. This includes input from other cholinergic interneurons which is mediated through excitatory nicotinic acetylcholine receptors and inhibitory M2 autoreceptors (Lim et al., 2014). The majority of efferents of cholinergic interneurons run to MSNs. Their functioning is regulated through binding muscarinic receptors. GABA-ergic interneurons are inhibited through M2 receptors (Lim et al., 2014). The same is true for thalamostriatal and corticostriatal glutamatergic terminals. However, these glutamatergic terminals may, similarly to dopaminergic terminals, also be stimulated by nicotinic receptors (Lim et al., 2014). The cerebral distribution of acetylcholine muscarinic M1–M5 receptors has been elucidated using immunological techniques (Hersch et al., 1994; Levey et al., 1991). All five subtypes have been found, but the vast majority of the total solubilised muscarinic binding sites of the rat brain consists of M1, M2 and M4 receptors. Light and electron microscopic immunocytochemistry revealed that, within the rat striatum, 78% of the neurons express muscarinic M1, 44% of the neurons M4 and only 2.5% express M2 receptors. M3 receptors are present in a subset of spiny dendrites (of MSNs). M2 receptors are predominantly muscarinic autoreceptors (Hersch et al., 1994). There is evidence that direct and indirect pathway MSNs are mutually differently affected by cholinergic interneurons due to differences in their expression of excitatory M1 and inhibitory M4 receptors (Bernard et al., 1992; Santiago and Potter 2001; Yan et al., 2001). Both direct and indirect pathway MSNs carry both types of muscarinic receptors, but while M1 receptors are equally distributed, inhibitory M4 receptors are far more abundant in direct pathway MSNs. Santiago and Potter (2001) have demonstrated that only 14% of M4 receptors were found on indirect pathway MSNs, and 86% on direct pathway MSNs. Hence, non-selectively blocking M1–M5 muscarinic receptors with biperiden or trihexyphenidyl (Cyclodol) (Bolden et al., 1992) will primarily lower the activity of indirect pathway MSNs by blocking spontaneously activated excitatory M1 receptors. This may explain, at least in part, their effectivity in Parkinsonism (and akathisia), because direct pathways increase and the indirect pathway decreases motor activity (Loonen and Ivanova, 2013).

Blocking muscarinic receptors non-selectively with anticholinergic drugs (Bolden et al., 1992) is common procedure in the treatment of antipsychotic drug-induced dystonia (Simon et al., 2017; Van Harten et al., 1999). Concomitant (prophylactic) anticholinergic treatment of extrapyramidal side effects is, however, also considered to be one of the risk factors for tardive dyskinesia (Jankelowitz, 2013), although Altamura et al. (1990) did not find a significant association between anticholinergic drug use and the prevalence of tardive dyskinesia. On the other hand, some studies suggest an improvement in tardive dyskinesia with the cessation of anticholinergics (Desmarais et al., 2012).

The pharmacological selectivity for muscarinic receptor subtypes of some antipsychotic and anti-Parkinsonian drugs is described by Bolden et al. (1992) and Bymaster et al. (2003). Anticholinergic agents are fairly potent (Kd≈1–10 nM), but have hardly any selectivity for specific muscarinic receptor subtypes (Bolden et al., 1992). The affinity of most antipsychotics is considerably lower than classical muscarinic antagonists (Bolden et al., 1992; Bymaster et al., 2003). Only clozapine and olanzapine have similar affinity for M1, M3, M4 and M5 receptors, but bind with somewhat lower affinity to M2 (Bymaster et al., 2003). In addition, some compounds are known to be partial agonists for specific subtypes (e.g. clozapine for M4) (Michal et al., 1999) and, in theory, future antipsychotic compounds could be allosteric muscarinic modulators (Yohn and Conn, 2018). The latter is, however, more often associated with potential therapeutic (cortical) effects than with extrapyramidal (basal ganglia) related side effects.

## The mechanism of drug-induced dystonia

Specifically focused research on the pathophysiology of drug-induced dystonia is certainly not an easy matter. Empirically, both acute and tardive forms are relatively scarce and unpredictable, which makes it difficult to prospectively select a patient population containing enough persons who will eventually experience such phenomena. For example, in a placebo- and haloperidol-controlled clinical trial of an antipsychotic drug in the treatment of 353 patients experiencing an acute exacerbation of schizophrenia conducted at 33 sites in the USA between August 2002–May 2003, only 16 patients (4.5%) suffered from dystonia (Potkin et al., 2015). Apart from their limited number, the geographical spread incapacitates practically the ability to rapidly include these patients in sophisticated experimental studies once dystonia has occurred. Moreover, the ethical requirement to obtain informed consent raises difficulties because persons with an acute psychosis suffering from intrusive side effects are not likely to agree with participation. The low incidence of acute as well as tardive antipsychotic drug-induced dystonia also limits the possibility to include sufficient patient numbers for specifically designed pharmacogenetic studies. Therefore, the strategy we have applied to elucidate the mechanisms of tardive dyskinesia is also not feasible here (Loonen et al., 2019). Moreover, it is difficult to isolate specific drug-treatment related factors that induce dystonic movements from more general aspects which increase the likelihood of experiencing such side effects like, for example, factors changing receptor concentrations of involved antipsychotic drugs. Therefore, a more hypothesis-based method to understand drug-induced dystonia is indicated utilising knowledge of the functional anatomy of movement and the physiopathology of other types of dystonia, and adding specific pharmacological findings or viewpoints. This strategy will be used in the present article.

Pharmacological themes which could be implemented are as described earlier: (a) efficacy of drugs antagonising muscarinic receptors in acute and, to a lesser extent, tardive dystonia; (b) the relationship of both types of dystonia with extensive, fluctuating activities of dopamine D2 receptors; (c) the increased vulnerability for acute dystonia induced by recent cocaine abuse; (d) the action mechanisms of drugs used to treat (other) dystonias like intrathecal baclofen (Bonouvrié et al., 2019) or botulinum A toxin; and (e) the distribution and expression of pharmacological targets in motor extrapyramidal circuitry (see introduction). A remaining problem is that dystonia can also occur during treatment with drugs other than antipsychotics (Jain, 2012; Van Harten et al., 1999) and that primary and secondary movement disorders have a heterogenous character (with different genetic, biochemical, neurophysiological and pharmacological aspects), which does not immediately suggest a single pathophysiological mechanism (Breakefield et al., 2008; Jinnah and Hess, 2018; Quartarone and Ruge, 2018). Striatal cholinergic dysfunction is a unifying theme in the pathophysiology of dystonia (Eskow Jaunarajs et al., 2015), but this is, of course, not independent from the efficacy of anticholinergic treatment. Stavrinou et al. (2011) have tried to create a unified dystonia pathophysiology model. Their simplistic model describes underlying defects of basal ganglia function which result in increased cortical excitability, misprocessing of sensory feedback and aberrant cortical plasticity. With important adaptations, this model can be applied to the explanation of drug-induced dystonia. Moreover, dystonia is best conceptualised as a motor circuit disorder rather than an abnormality of a particular brain structure (Tanabe et al., 2011).

### Role of converging extrapyramidal pathways

Cortical glutamatergic neurons projecting to the striatum are the first component of parallel convergent CSTC circuits. These corticostriatal terminals originate within all cerebral lobes, but within the striatum their terminals are not evenly distributed over striosomal and matrix compartments (Shipp, 2017). Within the continuous ‘matrix’ compartment these projections are more or less topographically arranged, although patchy cortico-striatal converging processing units can be distinguished, forming the start of these CSTC circuits (see Shipp, 2017). Some of the CSTC circuits starting within the frontal cortex are closed by projections via the basal ganglia to the thalamus and thereafter back to the frontal cortex. Within the complete set of CSTC circuits, glutamatergic input from all areas of the cerebral cortex ultimately converges to their relevant areas of the frontal cortex (Calabresi et al., 2007; Loonen and Ivanova, 2013), for example, corticostriatal projections from the somatosensory cortex, motor cortex, anterior cingulate cortex and supplementary motor area converge via the putamen and thalamus on a specific spot of this supplementary motor area (Alexander et al., 1986). Each CSTC circuit contains a direct pathway stimulating the frontal cortical target as well as an indirect pathway inhibiting the same target. Glutamatergic corticostriatal and thalamostriatal fibres stimulate MSNs of the direct (which augments the movement ) and indirect (which decreases the movement ) pathways of the same CSTC circuit differently, and this allows exact regulation of the speed and intensity of the necessary activation of specific frontal cortical areas. This extrapyramidal regulation is added to glutamatergic intracortical connectivity which affects the same frontal cortical site. During the training of cognitive and motor tasks, the activity of the involved glutamatergic synapses is adapted by LTP and LTD of the involved glutamatergic synapses to the correct level in order to allow proper execution of the task. Neuroplastic changes within other components of the regulatory network are also involved in this training process and may a play a role in the induction of dystonic movements.

### Somatosensory to motor processing

Perhaps consideration of the pathophysiology of focal dystonia such as task-specific focal dystonia (Altenmüller and Müller, 2013; Goldman, 2015; Hallett, 2011) and cervical dystonia (Brugger et al., 2019; Popa et al., 2018) can help to understand the mechanism of antipsychotic drug-induced dystonia. Both types of focal dystonia may also show transient improvement when applying so-called ‘sensory tricks’ (Mailankody and Pal, 2017), both types may respond to chemodenervation with intramuscular botulinum toxin injection in relevant muscles (Kaňovský and Rosales, 2011; Rosales and Dressler, 2010) and both are accompanied by altered proprioceptive functioning (Mailankody and Pal, 2017; Nevrlý et al., 2018). This indicates that aberrant functioning within somatomotor circuits may be a common principal physiopathological mechanism. In some types of dystonia, dysfunction of the cerebral cortex (Altenmüller and Müller, 2013; Brugger et al., 2019; Hallett, 2011) or cerebellum (Popa et al., 2018; Prudente et al., 2014) may be most important, but in antipsychotic drug-induced dystonia this more likely to represent a malfunctioning of converging CSTC circuits within the basal ganglia. This is supported by the close relationship of drug-induced dystonia with a blockade of dopamine D2 receptors and the increased vulnerability to acute drug-induced dystonia after exposition to cocaine. Dopamine has a much larger neurotransmitter role within the basal ganglia than within the cerebellum or somatosensory and motor cerebral cortices. Chronic excessive cocaine usage results in decreased dopamine signalling both within ventral and dorsal striatum (Willuhn et al., 2014). At this stage, the acute antidopaminergic effects of D2-antagonists are aggravated by the prior decreased dopaminergic signalling. This would enhance the propensity for acute dystonia. Persons who abuse cocaine often use excessive dosages regularly.

### Role of neuroplastic modulation

Aberrant plasticity and learning may have a role in causing (non-drug-induced) dystonia (Avchalumov et al., 2014; Mink, 2018). This hypothesis could offer a link to the mechanism of antipsychotic drug-induced dystonia. Affecting dopaminergic neurotransmission within the basal ganglia may induce neuroplastic alterations within direct and indirect pathway CSTC circuits (Calabresi et al., 2007). An important form of neuroplasticity is accomplished by inducing an enduring change in sensitivity of the glutamatergic synapses due to LTP (Brown et al., 1988; Loonen, 2013) and LTD (Bashir, 2003; Loonen, 2013). Stimulation of dopamine receptors affects both of these (Calabresi et al., 2007; Centonze et al., 2003b; Lovinger, 2010) and neuroplastic changes are also known to have an important role in inducing cocaine addiction (Lee and Dong, 2011; Thomas et al., 2008; Willuhn et al., 2014). Excessive dopamine release which results from abusing cocaine can, possibly, induce acute neuroplastic changes by stimulating dopamine D1 and D2 receptors (Calabresi et al., 2007) and these could destabilise the extrapyramidal regulatory system. It is possible to think that such a mechanism would explain why recent exposure to cocaine results in proneness to dysregulation in a dystonic manner. LTP/LTD are also affected by muscarinic cholinergic transmission (Calabresi et al., 2007; Centonze et al., 1999, 2003b; Lovinger, 2010). Cholinergic M1 type receptors play a crucial role in LTP (Centonze et al., 1999, 2003b) and, through an indirect mechanism, also affect LTD (Calabresi et al., 2007). However, this mechanism is unlikely to be involved in causing proneness to dystonia or their prophylactic activity in acute dystonia.

### Dopamine D2 receptor blockade

Antagonism of dopamine D2 receptors is, until now, the foremost characteristic of antipsychotic agents. Antipsychotic drug-induced dystonia is obviously related to this pharmacodynamic property, which is especially true for its acute type as this appears earlier in time and with lower dosages when dopamine D2 affinity of the drug is higher (Ayd Jr, 1961). Dopamine D2 antagonism combined with relative sparing of dopamine D1 receptors is likely to result in a disbalance between the activities of direct and indirect extrapyramidal pathways. This would first of all result in Parkinsonism which is characterised by hypokinesia and rigidity. However, the execution of well-trained motor programmes requires harmonised activity of converging and parallel components of CSTC circuits. In acute antipsychotic drug-induced dystonia, the sudden increase of the activity of indirect pathways can disturb the coordinated pattern of CSTC circuit activation. In tardive dystonia the variable disinhibition of indirect CSTC pathways – variability which is typical for drug treatment-induced processes – could possibly play a role. Tardive dystonia has a gradual delayed onset and usually continues after the usage of the antipsychotic drug has been ended. Pharmacokinetically-induced fluctuating disinhibition of striatal indirect pathway MSNs could result in a maladjustment of CSTC activities during the continuous training which is necessary for a proper execution of complex motor programmes. The use of anticholinergic medication could decrease the influence of an improperly functioning CSTC circuit. These anti-Parkinsonian drugs are non-selective muscarinic receptor antagonists (Bolden et al., 1992; Bymaster et al., 2003) which actually denervate the tonically active cholinergic interneurons by blocking muscarinic M1 as well as M2-class receptors affected by their terminals. This denervation could result in a global decrease of the influence of the entire extrapyramidal system, hence the importance of the trained pattern of activation during the execution of motor programmes. In this respect, blocking excitatory M1 class (M1, M3, M5) muscarinic receptors is estimated to be more important than antagonising inhibitory M2 class (M2, M4) receptors. Excitatory M1 receptors are present on both direct and indirect pathway MSNs, while inhibitory M4 receptors are far more abundant in direct pathway MSNs (Santiago and Potter 2001). In direct pathway MSNs, blocking stimulation by activating M1 receptors could easily overrule disinhibition by blocking M4 receptors. This mechanism may, at least partly, also explain why clozapine has beneficial effects in the treatment (and prevention) of tardive syndromes (Hazari et al., 2013). Clozapine is a partial agonist on muscarinic M4, and probably also M2 receptors (Michal et al., 1999) next to its (antagonistic) binding to muscarinic M1, M3 and M5 receptors (Bolden et al., 1992). The combination of partial intrinsic activation of M4 receptors makes the functional elimination of both direct and indirect pathways even more effective with clozapine in comparison to anticholinergic antiparkinsonian drugs.

Presynaptic dopamine D2 receptors also inhibit the activity of parvalbumin-expressing fast-spiking interneurons by inhibiting the release of dopamine which stimulates postsynaptic dopamine D5 receptors which stimulate these interneurons (Bracci et al., 2002; Centonze et al., 2003a). Blocking these dopamine D2 receptors could – at least in theory – contribute to acute dystonia by massive activation of fast-spiking GABA-ergic interneurons, but this mechanism is not supported by the effects of benzodiazepines which increase GABA-ergic neurotransmission but in general do not worsen dystonia.

### Specific role of aspiny cholinergic interneurons

Apart from the above role of cholinergic interneurons in allowing the proper functioning of mutually relevant converging and parallel CSTC circuits, antipsychotics can also induce dystonic movements by affecting the activity of cholinergic interneurons directly. Striatal cholinergic interneurons receive afferent input with a wide variety of neurotransmitters (Lim et al., 2014), including glutamatergic input from especially the intralaminar thalamic nuclei (Assous and Tepper, 2019), nigrostriatal dopaminergic input (Lim et al., 2014) and cholinergic input from other cholinergic interneurons. Although dopaminergic projections to MSNs are by far more abundant than those on cholinergic interneurons (Sizemore et al., 2016), the majority of these interneurons express inhibitory dopamine D2 receptors next to excitatory D5 receptors, and about 20% contain low levels of excitatory D1 receptors (Lim et al., 2014). Nigrostriatal projections largely enhance the activity of certain CSTC circuits by affecting specific MSNs and modify the activity of a group of MSNs by inhibiting the more widespread activity of cholinergic interneurons. Via thalamostriatal projections from, in particular, the intralaminar thalamic nuclei (Bostan and Strick, 2018; Sciamanna et al., 2012; Smith et al., 2004), cholinergic interneurons play an essential role in integrating cortical and cerebellar neuronal networks by gating CSTC processing (Bostan et al., 2018; Ding et al., 2010; Sciamanna et al., 2012). This is probably mediated by enhancing muscarinic M1 and M4 receptor activation which interrupts cortical signalling to striatal MSNs (Bordia and Perez, 2019; Sciamanna et al., 2012).

## Conclusion

Antipsychotic drug-induced dystonia is probably related to the dysregulation of neuronal networks which are involved in processing somatosensory activity in order to generate a sophisticated complex motor response (Table 3). Proper execution of the required motor programme depends upon adequate functioning of a set of parallel and converging extrapyramidal CSTC circuits. Through training of how to execute a complex movement, the activities of the CSTC circuits are adapted by neuroplastic processes. Via intralaminar thalamostriatal projections and striatal aspiny cholinergic interneurons as a ‘common’ pathway, both the CSTC and the cerebellar neuronal networks can modulate the sensitivity of striatal MSNs to cerebrocortical input. This mechanism leads to integration of cerebrocortical and cerebellar input during the execution of the individual’s complex movements. Indirect pathway MSNs, as well as cholinergic interneurons, predominantly express inhibitory dopamine D2 receptors, and their sudden nonselective disinhibition after the initiation of treatment with a relative high dosage of a dopamine D2 antagonist (for example, intramuscular injection of a potent antipsychotic drug) may distort proper execution of the well-trained motor programme in acute dystonia. Prior exposition to the effects of a high dosage of cocaine may increase the vulnerability for this dysregulation, since excessive cocaine intake leads to the massive release of dopamine, and this may prime the neuronal system. Neuroplastic changes may play an important role in causing tardive dystonia. Fluctuating disinhibition of indirect pathway MSNs and cholinergic interneurons during the execution of complex motor programmes may induce adverse neuroplastic changes which could result in maladjustment of the activity of CSTC circuits during the execution of complex motor programmes. The modification by the tonically active giant aspiny cholinergic interneurons could explain the therapeutic effects of anticholinergic drugs. The involvement of (possibly) specifically muscarinic M1-type receptors indicate putative advantages of selective muscarinic M1 antagonists. Moreover, our hypothesis indicates that modulating the vulnerability to neuroplastic changes within indirect pathway MSNs could have a prophylactic potential.

**Table 3.** Summary of the putative background of antipsychotic drug-induced dystonia.

*Mismatch of activity of direct and indirect CSTC pathways* Relationship of dystonia with dopamine D2 receptor blockade Increased vulnerability to develop acute dystonia by recent cocaine abuse Relationship between neuroplastic alterations and D2 receptor blockade Efficacy of treatment with muscarine receptor antagonists
*Mismatch of convergence of CSTC circuits* Analogy with primary focal dystonia Widespread distribution of the influence of single cholinergic interneurons Neuroplastic mechanism of training for the proper execution of motor tasks Involvement of cholinergic interneurons in cerebrocortical and cerebellar input integration Putative role of disinhibition of cholinergic interneurons in acute dystonia

CSTC: cortical-striatal-thalamic-cortical.
